# Integrating the quantitative with the qualitative: findings from a mixed methods cardiac rehabilitation exercise trial

**DOI:** 10.1016/j.hroo.2024.06.003

**Published:** 2024-06-08

**Authors:** Oonagh M. Giggins, Suzanne Cullen-Smith, Eanna Kenny, Julie Doyle

**Affiliations:** ∗ NetwellCASALA, Dundalk Institute of Technology, Dundalk, Ireland; † Health Behaviour Change Research Group, School of Psychology, University of Galway, Galway, Ireland

**Keywords:** Cardiovascular disease, Cardiac rehabilitation, Exercise, Remote rehabilitation, Mixed methods trial

## Abstract

**Background:**

Cardiac rehabilitation is a core component of cardiovascular disease management. Eastern Corridor Medical Engineering–Cardiac Rehabilitation is a digital health platform for online cardiac rehabilitation exercise. We conducted a mixed methods pilot trial to evaluate Eastern Corridor Medical Engineering–Cardiac Rehabilitation.

**Objective:**

The study sought to examine the difference between objectively measured outcomes and participant perceptions of benefits and improvements gained from participation in a cardiac rehabilitation exercise program.

**Methods:**

Seventeen participants (14 male, 3 female; 69.5 ± 7.3 years of age) took part and were allocated to 1 of 2 groups; an online exercise group (n = 8), or an in-person exercise (n = 9) group. Due to the COVID-19 pandemic, a pragmatic approach to group allocation was adopted. Objective outcomes were assessed at baseline and repeated following the intervention period, with the primary outcome being 6-minute walk test distance. In addition to clinical outcome measurements, we undertook qualitative interviews with participants.

**Results:**

Only 5 participants demonstrated a clinically meaningful improvement in 6-minute walk test distance, following the 8-week exercise program. The main theme emerging from the qualitative interviews was the valued benefits of the cardiac rehabilitation exercise program. Despite the lack of measurable physical change, participants self-defined a range of benefits they valued and attributed directly to participation in the cardiac rehabilitation exercise program.

**Conclusion:**

The findings from this study may offer a useful starting point for further study of community-based cardiac rehabilitation exercise and also highlight the benefit of adopting a mixed methods approach that considers both the objective outcomes measured as well as the subjective reports obtained from participants.

## Graphical abstract

**Figure:**
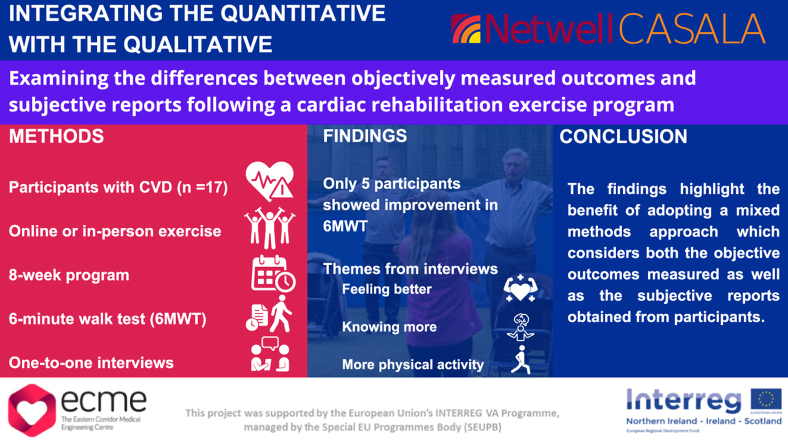

**Key findings**

▪ A mixed methods pilot trial was carried out to assess Eastern Corridor Medical Engineering–Cardiac Rehabilitation, a digital health platform for the remote delivery of cardiac rehabilitation exercise.
▪ No significant changes were observed in the objective outcome measures were at the 8-week follow-up.
▪ However, qualitative reports suggested that participants felt fitter and stronger after the program.
▪ The findings emphasize the advantages of using a mixed methods approach.

## Introduction

Cardiovascular disease is a major global health challenge and leading cause of death worldwide. Cardiac rehabilitation is recognized as a core component of cardiovascular disease management and is an established recommendation for all patients following an acute cardiac event, to prevent further illness and mortality.^1^, ^2^, ^3^ The benefits of cardiac rehabilitation have been reviewed extensively. Exercise-based cardiac rehabilitation has been shown to reduce the risk of myocardial infarction, all-cause mortality, and all-cause hospitalization, as well reduce associated healthcare costs.^4^

New models of cardiac rehabilitation using information, communication, and digital technologies have been suggested to improve access and increase participation in cardiac rehabilitation. These models deliver cardiac rehabilitation directly into the person’s home and gained significant traction during the COVID-19 pandemic when many in-person cardiac rehabilitation programs were suspended.^5^, ^6^, ^7^ Remotely delivered cardiac rehabilitation may represent a more convenient, flexible, and attractive alternative to people with cardiovascular disease than the traditional center-based rehabilitation. We developed a digital health platform, Eastern Corridor Medical Engineering-Cardiac Rehabilitation (ECME-CR), to support the online delivery of cardiac rehabilitation exercise.

A mixed methods pilot trial was conducted to examine the feasibility of delivering an online cardiac rehabilitation exercise program supported by the ECME-CR platform. This trial also compared outcomes between a group of participants undertaking the online exercise program with those in a control group who participated in a traditional in-person exercise class. In addition to clinical outcome measurements, we undertook qualitative interviews with all participants enrolled in the trial. The quantitative pre-post change in clinical measures outcomes were analyzed and reported separately.^8^ Statistically significant improvements in the outcome measures used were not observed at the 8-week follow-up; however, qualitative reports by participants indicated widespread satisfaction with participating in the cardiac rehabilitation exercise program, both online and in person. To generate further insights about the intervention, the quantitative outcomes and qualitative data from this mixed methods trial were integrated.^9^ In this article, we jointly display the quantitative outcomes with participant qualitative reports, to examine the differences between the objectively measured outcomes and participant perceptions of outcomes gained from participation in the cardiac rehabilitation exercise program. Further, data from both intervention and control groups are combined, as comparison of both groups has been conducted elsewhere,^8^ and the purpose of this article is to supplement the objective outcome data with the subjective participant reports.

## Methods

### Study participants

The study population included participants with a variety of cardiovascular disease histories, who were eligible to participate in phase IV (community-based) cardiac rehabilitation. The study protocol was reviewed and approved by the Human Research Ethics Committee in Dundalk Institute of Technology. The research reported in this article was conducted in accordance with the Declaration of Helsinki. All participants provided written informed consent before entering the study.

### Cardiac rehabilitation exercise program

After providing informed consent, participants were assigned to either an intervention group (online exercise) or a control group (in-person exercise). Both study groups performed the same cardiac rehabilitation exercise program over an 8-week intervention period. The exercise program consisted of twice-weekly exercise classes, with each class lasting 1 hour. Details of the exercise program are outlined elsewhere.^10^ Participants in the online group partook in the exercise program in their own home, while the control group attended their exercise classes in a sports center in the institution.

### Eastern Corridor Medical Engineering–Cardiac Rehabilitation

The ECME-CR digital health platform was designed by the ECME research team at NetwellCASALA, Dundalk Institute of Technology, to support the virtual delivery of cardiac rehabilitation exercise. The platform consists of a responsive Web-based app (ECME-CR app), digital devices, and the CABIE+/SIMS system and is described fully described elsewhere.^10^ Two off-the-shelf consumer digital devices, the Withings ScanWatch and BPM Connect (Withings) are integrated with the platform and are used to collect health and well-being data (heart rate, blood pressure, physical activity). Participants can view this data on the ECME-CR app. CABIE+ is the data collection and aggregation system that organizes and stores the data acquired from the ECME-CR app and the integrated Withings’ devices, while SIMS is the information management system that allows the exercise instructor/research team to view, analyze, and interpret the data collected from the application and the devices in close to real time for individual participants. The design of the ECME-CR app was informed by learnings from previous research, including interviews and co-design sessions conducted with older adults with cardiovascular disease.^11^^,^^12^

Participants in the online group partook in the exercise program in their own home using the ECME-CR app during each class, with the classes delivered via Zoom videoconferencing.

### Data collection

Quantitative outcome data were collected at baseline (T1) and were repeated at the end of the 8-week intervention period (T2). Participants attended the research center for outcome measurements. The primary outcome measure was cardiopulmonary exercise capacity, assessed using a 6-minute walk test.^13^ The 6-minute walk test was performed as per the European Respiratory Society/American Thoracic Society’s guidelines,^14^ using a 30-m-length walking course. Participants walked back and forth along the walking course, covering as much distance as possible in 6 minutes. The test was performed twice to account for a learning effect,^12^ and the longer distance was recorded. A 25-m improvement in the 6-minute walk test distance is considered clinically meaningful.^15^

Secondary outcome measures included measurement of self-reported quality of life via the 12-item Short Form Survey (SF-12).^16^ Two summary scores were calculated for the SF-12, the physical component summary and mental component summary scales. Higher physical component summary and mental component summary scores indicate better health related quality of life, a positive change indicates improvement, and a negative change indicates deterioration. A change of >3.8 in physical component summary and >3.3 in mental component summary is considered a clinically important difference in quality of life.^17^

All participants who completed the 8-week intervention were also invited to participate in a one-to-one interview at T2. The interviews were semi-structured and were undertaken either in person or via telephone. Interview questions were developed, and an interview schedule (Supplementary Appendix 1), comprising open-ended questions, was developed beforehand. Each interview was digitally recorded and transcribed verbatim.

### Data analysis

Six-minute walk test distance and physical and mental component summary scores collected at T1 and at T2 were collated using Microsoft Excel. Descriptive statistics (mean and standard deviation) were used to summarize participant characteristics. The difference (Δ) in 6-minute walk test distance and the physical component summary and mental component summary scores from T1 to T2 for each participant were calculated. Further statistical analyses were conducted to examine the mean differences in study outcome measures between groups (intervention group and control group) and to examine the impact of time, and the results of these analyses are presented elsewhere.^8^

A reflexive thematic analysis was conducted on the qualitative interview data, following the steps outlined by Braun and colleagues^18^ and Braun and Clarke,^19^ who posited that thematic analysis is a useful qualitative approach for use in applied research.^20^ Three authors (O.G., S.C.S., and E.K.) reviewed interview transcripts for initial data familiarization. Initial auto-coding by interview question topic was completed using NVivo (release 1.7.1) for Windows (QSR International). Two researchers (S.C.S. and E.K.) independently coded all interview transcripts using an inductive semantic coding approach. E.K. coded each transcript in its entirety from start to finish and S.C.S. coded the content by question topic and both sets of coding were compared. Agreement was reached where code differences arose, before an agreed codebook was created and tested against the dataset for stability. Next, the multidisciplinary project team consulted and agreed on potential latent meanings on the initial codes. Findings from this analysis are currently under review elsewhere. To examine the unanticipated discrepancy between objective results and subjective participant reports of improvement, a further iterative integrative mixed methods analysis of the data was then conducted, in line with the process outlined by Guetterman and James.^21^

For the integrative merging of qualitative and quantitative data, a description of whether each variable (6-minute walk test distance, physical component summary, and mental component summary) at T2 was better, worse, or the same as at baseline was used. These variables were imported into NVivo using an Excel spreadsheet. A series of interactive cross-tabulation matrices were run using the NVivo query function, for example to compare what was said about physical improvements by those whose SF-12 physical component summary had improved and those whose SF-12 physical component summary had disimproved at T2. A sample of 11 transcripts were re-examined, from start to finish, by S.C.S., who reviewed and revised codes and constructed initial themes relating to the focus of this article. Following further interrogative discussion, final themes and subthemes (both semantic and latent) were refined. The process examined patterns and relationships between codes or across quantitative clusters, from a multidisciplinary perspective. This contributed to the systematic development of in-depth qualitative themes, thereby supporting the development of meta-inferences, or conclusions drawn from the merged qualitative and quantitative datasets and avoiding simplistic quantifying of qualitative data (counting coded references) as a means of data integration.^22^

## Results

### Participants

A total of 21 participants with cardiovascular disease enrolled in the trial; 11 were allocated to the intervention group (online exercise) and 10 were allocated to the control group (in-person exercise). Two participants in the online exercise group withdrew during the intervention period, due to family reasons and changing work commitments. One participant in the in-person exercise group withdrew from the study, prior to baseline measurements, due to a lower limb injury. One further participant in the online exercise group was unable to attend the T2 measurement sessions due to personal reasons. In all, 8 participants (male: n = 5; 69.7 ± 7.2 years of age; height 163.9 ± 5.4 cm; weight 81.6 ± 14.1 kg) in the online group and 9 participants (male: n = 9; 69.8 ± 8.2 years of age; height 173.8 ± 5.2 cm; weight 94.4 ± 18.0 kg) in the in-person group completed the exercise program, the T2 interview, and both measurement sessions. All interviews were conducted in person. One participant in the intervention group was unable to perform the 6-minute walk test at T2 due to a rheumatic flare-up, and therefore, no 6-minute walk test data are presented for this participant. Participants had a variety of cardiovascular disease histories, with 12 participants having undergone a percutaneous coronary intervention, 2 undergoing coronary artery bypass grafting, 1 undergoing an aortic valve replacement and percutaneous coronary intervention, 1 undergoing a percutaneous coronary intervention and coronary artery bypass grafting, and 1 being treated with medication only during their hospitalization. Most participants were engaging in some form of physical activity at baseline prior to enrolling in the exercise program (Table 1).

**Table 1:** Participant characteristics (N = 17)

Age, y	69.5 ± 7.3
Sex	
Female	3
Male	14
Cardiovascular disease history	
Percutaneous coronary intervention	14
Coronary artery bypass grafting	3
Aortic valve replacement	1
Medication only	1
Physical activity behavior	
None	5
Light-intensity physical activity	1
Moderate-intensity physical activity	11

Values are mean ± SD or n.

### Quantitative outcomes

The difference (Δ) in 6-minute walk test distance and the SF-12 physical component summary and mental component summary scores from T1 to T2 for each participant are presented in Table 2. Regarding the 6-minute walk test distance, only 5 participants demonstrated a clinically meaningful improvement (positive Δ) from T1 to T2 (Table 2). Additionally, for the SF-12 physical component summary score, 3 participants demonstrated clinically meaningful improvements in physical health related quality of life at T2 (Table 2). Five participants demonstrated clinically meaningful improvements in the mental component summary score of the SF-12 from T1 to T2 (Table 2). Perceived improvements, attributed to the cardiac rehabilitation exercise program participation, were coded within the interview data and are identified for each participant in Table 2.

**Table 2:** Joint display of participant quantitative outcomes and summary of perceived outcomes

	6MWT distance (m)			SF-12 physical component summary			SF-12 mental component summary			Perceived physical, psychological, behavioral improvements
	T1	T2	Δ	T1	T2	Δ	T1	T2	Δ	
P2	464.8	429.8	–35	34.91	46	11.1	59.77	43.3	–16.47	Increased confidence in exercise capability Learned about pacing during exercise Learned about exercising and physical movement Physical improvement
P3	406	427.9	21.9	53.97	41.2	–12.8	58.83	63.5	4.67	Learned about exercising and physical movement
P4	365.9	384.1	18.2	27.74	41.5	13.8	64.04	60.6	–3.44	Increased confidence in exercise capability Physical and psychological improvement
P5	530.2	527.4	–2.8	55.5	55.5	0.0	57.8	57.8	0	Enjoyed participating but no benefits identified
P6	585	645	60	38.7	44.8	6.1	59.6	55.7	–3.9	Learned about pacing during exercise Physical improvement Increased daily physical activity
P7	538	540	2	44.8	34.5	–10.3	56.2	61.9	5.7	Physical improvement Increased daily physical activity
P8	540.4	577	36.6	56.2	54.5	–1.7	47.6	57.1	9.5	Physical and psychological improvement Motivated to do more exercise since CRE program
P9	No data	No data	No data	38.9	16.9	–22.0	59.4	51.2	–8.2	Increased awareness about monitoring blood pressure
P10	505	540	35	50.1	50.4	0.3	35.2	56.9	21.7	Physical improvement
P11	465	477.7	12.7	54.8	53.6	–1.2	59.8	60.8	1	Motivated to do more exercise since CR class
P12	511.5	528.3	16.8	51.5	44.8	–6.7	57.4	58.3	0.9	Psychological improvement
P13	561	547	–14	50.9	53.4	2.5	55	55.2	0.2	Learned about pacing during exercise Physical improvement
P14	570	601	31	43.7	40.3	–3.4	53.8	61.7	7.9	Learned about exercising and physical movement
P18	439.4	428.3	–11.1	54.8	54.8	0.0	59.8	59.8	0	Increased confidence in exercise capability Learned about pacing during exercise Physical improvement
P16	468.2	548	79.8	45.1	35.1	–10.0	55.8	55.5	–0.3	Physical improvement
P15	502	494.4	–7.6	44.6	29.2	–15.4	53	51.3	–1.7	Learned about exercising and physical movement
P17	570	533.1	–36.9	52.1	52.9	0.8	56.8	54.4	–2.4	Enjoyed participating but no benefits identified

The quantitative outcomes are presented as the difference (Δ) measured from baseline (T1) to the end of the intervention period (T2). Minimal clinically important differences: 6MWT = 25 m^16^; SF-12 physical component summary = 3.8; SF-12 mental component summary = 3.3.^17^

6MWT = 6-minute walk test; CR = cardiac rehabilitation; CRE = cardiac rehabilitation exercise; P = participant; SF-12 = 12-item Short Form Survey.

### Explanatory qualitative findings

The theme of the valued benefits of the cardiac rehabilitation exercise program demonstrated that despite the lack of measurable physical change, participants self-defined a range of benefits they valued and attributed directly to participation in the cardiac rehabilitation exercise program. Three related subthemes, feeling better (physically and psychologically), knowing more, and sustaining physical activity, comprised this overall theme and are discussed subsequently. The themes presented here do not represent an exhaustive list from the dataset, but rather represent those relevant to the question of what benefits and improvements participants perceived to have gained from taking part in the cardiac rehabilitation exercise program. Each theme is outlined in this section with reference to the related quantitative data outputs as appropriate.

### Valued benefits of the cardiac rehabilitation exercise program

All participants, except for 2, reported during their one-to-one interview that they had perceived to have improved in some way because of their participation in the cardiac rehabilitation exercise program. The improvements were both physical and psychological. In addition, participants reported gaining new knowledge as a benefit from participation.

#### Feeling better

Clinically meaningful improvement in 6-minute walk test distance and SF-12 scores were observed in only a few participants. However, physical benefits reported from participation included a perceived ability to do more activity than before participating in the cardiac rehabilitation exercise program. Participants perceived they could walk further or for longer:

“Well, I am more fit. I mean I have never been a fit person; I have never been an unfit person, but I am able to walk, without breathlessness, long distances now.”

This was, at times, despite a negative change in 6-minute walk test distance at follow-up. For example, this participant’s 6-minute walk test had not improved from T1 to T2, despite an assertion of greater fitness since participating in the cardiac rehabilitation exercise program. One subjective evaluation of improved fitness lay in perceptions of how physically robust participants felt while engaging in physical activity, either at preprogram levels, or in doing new activities learned during the cardiac rehabilitation exercise program. A specific subjective metric used was the extent of breathlessness while exercising, with a perceived reduction in breathlessness considered a physical improvement by a number of participants,

“I probably am walking, you know, a little bit easier...Like, you know, the odd time I’d go up to the shop to get the paper, I wouldn’t be as huffing, as puffing.”

Other subjective physical improvements reported included improvements in posture or balance. Specific elements of the cardiac rehabilitation exercise program were designed to address these, and some participants recognized these exercises as informative, effective, and easy to continue practicing at home: “I am doing more exercises now. I am getting up in the mornings and I am doing a few wee [small] weights.” It is notable that changes, whether physical, behavioral, or psychological, were often defined as small or moderate changes.

Overall, minor physical changes were linked together and presented, collectively, as evidence of sufficient progress to be considered satisfactory,

“I do feel, sort of, maybe a bit…. bad terminology but toned or I feel better, and I think I am standing probably straighter as well.”

“I think, I feel stronger you know. Physically stronger. Again, the breathing, the moving around. I mean I don’t slouch even if I am going up the stairs now I can, kind of, bound up a lot better than I did before.”

One participant whose T2 SF-12 physical component summary score trended toward improvement identified that the cardiac rehabilitation exercise classes were challenging but participation in-person encouraged greater exertion than he would have done on his own, and this motivational aspect of the program was seen as both positive and beneficial:

“I kind of struggled with that and I thought, yes, I need to get through this you know. It is like a mental push and, when I got to the end, I mean I could see myself that I was doing it quicker and enjoying it more... You are pushing yourself maybe that wee [little] bit harder. Whereas if you are on your own you might just slack a wee [little] bit.”

This participant, however, also quit smoking once enrolled in the trial and attributed some improvement to this overall commitment to health behavior change.

For those who reported no physical improvement, a lack of change was attributed to their pre-existing level of health or physical activity before participating: “It didn’t really improve with the physical fitness. It might have improved someone who didn’t do any exercises.” Furthermore, it may have been presumed that an existing level of fitness was required for participation (“I think I was fit enough to be able to take it [the cardiac rehabilitation exercise program] on”), and marked improvement of fitness may not have been the intended objective of participating.

The benefits of taking part in the cardiac rehabilitation exercise program went beyond the reported physical improvements of being able to walk further distances and feeling less short of breath on exertion, to include awareness of psychological and emotional benefits. Improved mood was reported as a result of participating in the cardiac rehabilitation exercise program: “When I started the program I think I would have said that I was a bit down on myself at times. Now I can’t say that now, I feel that there is a lift in me.” Objectively, this participant’s SF-12 mental component summary score had improved from T1 to T2. Participants reported enjoying engaging with others, including the class instructors and research team. The COVID-19 pandemic was ongoing at the time the cardiac rehabilitation exercise program was delivered, so in addition to providing an opportunity for social interaction, participation also provided a defined activity requiring both effort and commitment, both of which were valued by participants: “I enjoy …deep down I enjoy. I might complain a bit about it but I did enjoy it, you know. It gave me something to get up in the morning for, you know.”

#### Knowing more

The second subtheme, knowing more, included learning the value of monitoring physical symptoms and vital signs, such as blood pressure: “I am more aware of things, you know what I mean? It makes me more aware of your blood pressure how important it is to keep track of it and generally monitoring your heart, you know?” This participant did not identify any health or well-being improvements from participating but valued the knowledge acquired about monitoring his blood pressure, as a gain from participating in the program. Likewise, P3 identified learning about the potential cardiac strain from intensive swimming as a significant learning: “I didn’t think that swimming was so severe on your heart, as regards it was putting a lot of pressure on it.” For some participants, pain due to musculoskeletal issues was a lived challenge. Gaining the knowledge and skills to modify or adapt exercises to accommodate physical challenges, such as joint pain or mobility limitation, was a benefit of participation reflected in participant comments. One participant, who already exercised daily since their cardiac event 2 years previously, identified joint improvement following participation in the program:

“I have a full knee replacement on my right knee, and it is giving me awful trouble, because of the foot on the right and the stroke hit me on the right-hand side... My knee is far better from these classes than it was before I started, now.”

For others, learning how to adapt the type and intensity of exercise was necessary when managing pain, “I could reduce them [exercises] on other days and I was setting my pace.”

This acquired knowledge, in turn, informed or reinforced the position held by most participants, that exercise should be undertaken in moderation. Indeed, moderate-intensity exercise and avoiding overexertion was valued and was perceived as being reinforced or, indeed, recommended by the class instructors who advocated “pacing” while exercising. Fear of having another cardiac event, from overexercising, was a concern for some, which contributed to avoiding exercise in the past, but participation in the program showed participants how to exercise safely: “If you need a rest, take a rest. So, you weren’t really afraid of it [exercise]. If you needed to sit down, sit down and that was it, you know?” Encouragement from the class instructors, to avoid overexertion, resulted in increased confidence in participants’ ability to exercise.

#### Sustaining physical activity

Increased motivation to continue exercise and engage in physical activity was a further positive outcome for some participating in the cardiac rehabilitation exercise program. One participant, whose cardiac event had taken place within the previous year, while recognizing he was “still healing whereas they [other participants] are not,” was highly motivated to maintain and increase his physical activity. However, for most participants, their most recent cardiac event had taken place more than 5 years before they enrolled in this trial, with some as long as 20 years previously. Many had, therefore, already settled into a regular exercise and physical activity routine. Furthermore, as noted previously, reasons for participating in the program were generally not related to a desire to increase physical activity specifically, and motivation to change exercise behaviors was not strongly identified. Nonetheless, comparison with others was identified as a motivating factor during the exercise classes: “I am speaking about me, but I am looking at the other guys out of the side of my eye. You know we could have done a little bit more sprints, running, a little bit more exertion.”

Despite positive feedback from the program, those who had been engaged in limited exercise before participating acknowledged the intention to continue with exercise more, but some admitted that this may not be achieved. Reasons for this included being “busy” (“I would love to but…I am so busy doing nothing and I am very, very busy with everything, I never put a structure into my life.”) or not having an established support network with which to engage in physical activity (“I would like to do more of it but, again when I am on my own it’s difficult to motivate yourself.”). Nonetheless, the arrival of better weather was considered to be important in increasing physical activity: “Well the weather is better. I am out and about more. Gardening-wise, the hour in the evening and so on.” For 2 participants, the digital tools used during the cardiac rehabilitation exercise program provided incentive to maintain new physical activity levels, with an intention to purchase the monitoring devices or use exercise apps or videos. For those already engaged in regular physical activity, most planned to continue as before the program (“Ever since my operation, even before my heart attack I used to walk every night with another man.”) and considered this activity level sufficient (“I do the Aqua-aerobics on Monday, Wednesday, Friday and then the Tuesday morning and that’s, to my mind, with my condition and my age, I think I am doing great.”).

## Discussion

In this article, we have supplemented the quantitative objective outcomes from participants taking part in a cardiac rehabilitation exercise program with the subjective outcomes reported during the one-to-one interviews conducted at the end of the program. Exercise capacity was the primary objective outcome measure in this trial. Clinically significant improvements in 6-minute walk test distance, the measurement of exercise capacity, from T1 to T2, were observed in only 5 of the 17 participants completing the exercise program. However, the subjective reports obtained from most participants during their interviews indicted that they felt physically fitter, stronger, and able to do more than before. In addition to the perceived improvements in physical capacity, participants in this study also reported psychological improvements following their participation in the 8-week exercise intervention. Behavioral changes were also reported, such as increasing daily physical activity and a desire to maintain this after the completion of the program.

Overall, the experience of taking part in this trial was positive. The knowledge gained about exercise and their capacity for exercise were identified as further positive outcomes by many from participating in this trial. Improved knowledge was a benefit of cardiac rehabilitation also identified by others taking part in a virtual program.^23^ Many participants in this current trial realized that they were capable of doing more than they anticipated. Furthermore, participants appreciated the ability to adopt a more moderate approach to exercise during the cardiac rehabilitation exercise classes.

For most, continuing with their preprogram exercise or physical activity regime was planned, or the intention was expressed to incrementally increase physical activity, or undertake activities such as stretching or joint mobility exercises. This is similar to other studies in which participants reported greater motivation to manage their health condition and adopt healthier lifestyles as a result of their participation in a virtual cardiac rehabilitation program.^23^ Two participants in this study, however, admitted that they would probably not maintain a regular exercise regime after completion of the program.

Previous studies have indicated that barriers to participation in cardiac rehabilitation exercise programs can include physical disability, depression, poor social support, lack of physician referral, distance, and work or family obligations.^24^ For some in this trial, physical limitations or comorbidities presented challenges to completing some of the exercises, but these limitations did not present as a barrier to full engagement with the exercise program, as exercise modification was provided and encouraged by the class instructors. The importance of creating a safe and encouraging environment for cardiac patients during exercise has been outlined in previous studies.^25^^,^^26^ Participants in this trial reported feeling supported as they were exercising, which may have resulted in increased confidence and self-efficacy. This may in turn have helped to create positive reports of physical and psychological improvements following their completion of the cardiac rehabilitation exercise program. For others, the use of digital technologies was a motivating factor for physical activity. This is contrary to findings from others which indicated that many participants expressed skepticism or indifference in the application of digital technologies for enhancing physical activity.^27^

A previous cardiac rehabilitation trial that adopted a mixed methods approach demonstrated matched positive findings from the integration of quantitative and qualitative data.^26^ This current study is the first to our knowledge that found differences between the objectively measured outcomes and the participants’ subjective reports of their outcomes. These findings highlight the importance of considering both the objective and subjective outcomes of a trial. Participants in this study related a greater sense of control and competence over their cardiac rehabilitation exercise because of the support and encouragement received during the program, and this may have influenced their subjective reports. In addition, knowledge acquisition was identified as a positive contributor to participants’ perceptions about improvement following the cardiac rehabilitation exercise program, a finding supported by de Oliveira Nascimento and colleagues,^28^ who also found that their study respondents reported improved functionality.

While the findings reported here provide valuable insight, there are some limitations to the trial which should be acknowledged. First, the subjective reports from participants may have been subject to social desirability bias, in which participants gave responses considered more socially acceptable, rather than their actual beliefs or actions.^29^ Second, this trial included a homogeneous sample of predominately Caucasian men. Women and those from other ethnic cohorts may have different views, experiences, and outcomes from participating in cardiac rehabilitation exercise programs. Insights gathered from a review regarding patients’ personal cardiac rehabilitation experiences highlights that ethnic minorities continue to be at a disadvantage when it comes to accessing interventions, mainly related to cultural background, language challenges, and low physician referral rates,^30^ and therefore should be involved in future investigations. Women also face similar barriers to cardiac rehabilitation.^31^ Third, all participants in this study were self-referred, rather than physician referred, highly motivated, and willing to commit to completing the full program. In these respects, participants in this study may differ somewhat from previous cardiac rehabilitation studies showing poor completion rates.^32^^,^^33^ Furthermore, participants presented with a range of different cardiovascular diseases, as well as with a range of different comorbidities (such as joint arthropathy). However, the findings from this study may offer a useful starting point for further study of community-based, phase IV cardiac rehabilitation exercise. A fourth feature of this study that may be considered a limitation in the context of existing literature in the area may be the older age profile and length of time since most recent cardiac event represented among the participants. However, considering the aging of the population and the parallel increase in cardiovascular disease with both increasing age and multimorbidity, this study offers some real-world insights into relevant aspects for cardiac rehabilitation exercise program design and delivery for exploration in future research.

## Conclusion

To promote access and participation in cardiac rehabilitation, new models of cardiac rehabilitation have been proposed that make use of information, communication, and digital technology. This article compared the objective and subjective outcomes obtained in 2 groups of cardiovascular disease participants taking part in an 8-week cardiac rehabilitation exercise program. Participants in both the online and in-person exercise groups reported improvements in their physical or mental health and well-being following participation in the program. This was despite the fact that only few showed clinically significant improvements in objective outcome measures. These results demonstrate the value of using a mixed methods approach that takes into account both the subjective participant reports and the measured objective outcomes.
