# Supplementary material for: Integrating the quantitative with the qualitative: findings from a mixed methods cardiac rehabilitation exercise trial
Source: Heart Rhythm O2. 2024 Jun 8;5(7):443–51. doi: 10.1016/j.hroo.2024.06.003 (PMC11305875; doi:10.1016/j.hroo.2024.06.003)
Supplement: Supplementary file [file mmc1.doc]

| **Delivering Cardiac Rehabilitation Remotely using a Digital Health Platform** |
| --- |

**Welcome and Introduction**

Thank you for agreeing to participate in this interview. We are interviewing you to better understand your experiences of taking part in cardiac rehabilitation classes. We also want to learn how we could improve things for people with heart disease in the future. So there are no right or wrong answers to any of our questions, we are interested in your own experiences.

Participation in this study is voluntary and your decision to participate, or not participate, will not affect you in any way. The interview should take approximately one hour depending on how much information you would like to share. With your permission, I would like to audio record the interview because I don’t want to miss any of your comments. All responses will be kept confidential. This means that your de-identified interview responses will only be shared with research team members and we will ensure that any information we include in our report does not identify you as the respondent. You may decline to answer any question or stop the interview at any time and for any reason. Are there any questions about what I have just explained?

May I turn on the digital recorder?

*This guide only represents the main themes to be discussed with the participants and as such does not include the various prompts that may also be used (examples given for each question) Non-leading and general prompts will also be used, such as “Can you please tell me a little bit more about that?” and “What does that look like for you”.*

**Establishing Rapport**

Before we begin, it would be nice if you could tell me a little bit about yourself, your heart condition and how long ago you were diagnosed.

Tailor a question here to specific person and/or situation.

**Previous Experience with Cardiac Rehabilitation**

Can you tell me about your experience of CR before you enrolled in this study?

**Prompts:**

When did you take part - pre or post covid?

Where did the CR take place? Hospital/clinic based or virtually at home?

Was this your first time to participate in a CR programme?

What did you enjoy about taking part in CR exercise class? What did you not enjoy?

Did you experience any difficulties getting to/from the class (if hospital based)?

**Previous Experience with Technology**

Can you tell how much experience you had with technology before you enrolled in this study?

**Prompts:**

What devices do you own? Laptop/PC, smartphone, tablet device?

How often did you use these devices?

What did you use them for?

Did you use any videoconferencing platforms such as Skype or Zoom prior to enrolling in this study?

Did you take part in other online classes?

**Experience with Technology during study (for intervention group participants)**

How did you find the equipment familiarisation session?

**Prompts:**

Were the instructions given to you on how to operate the application, devices and how to join the virtual class useful?

Did the instruction manual provided assist you in understanding how to use the app/devices/join the class?

Did you feel confident in how to use the devices after the session?

Do you feel more confident with using technology as a result of your participation in this study?

Did you experience any technical difficulties during the virtual class?

**Prompts:**

How did you find joining the class via the videoconferencing platform?

How did you find interacting with the application/devices during the class?

**Experiences of Virtual Cardiac Rehabilitation (for intervention group participants)**

Can you tell me about your experience of taking part in the virtual cardiac rehabilitation class?

**Prompts:**

How did you get involved? Were you referred? By whom?

Let’s talk about your first virtual class. What stands out for you about that experience?

Was a friend or family member with you? How did you feel before joining the virtual class?

How would you describe your level of participation in the exercise classes? Did you find the classes engaging?

How would you describe the communication you had with the instructor? Were you able to interact with other class participants?

Was there anything you particularly liked about the virtual cardiac rehabilitation class?

For example, was it the exercises/the instructor/the group dynamic/being able to do it in your own home?

Was there anything you didn’t like about the virtual class?

Did you feel safe whilst exercising in the virtual class? Was a friend or family member with you during the classes?

The other group of participants in this study came into our gym twice a week for their exercise classes.

- What would your thoughts be on doing this type of exercise in a group in a gym or community centre?
- Do you think there are any benefits to doing these type of classes in a group in this setting?
- Do you think there are any drawbacks to doing these type of classes in a group in this setting?

**Experiences of In-Person Cardiac Rehabilitation Classes (for control group participants)**

Can you tell me about your experience of taking part in the cardiac rehabilitation exercise class?

**Prompts:**

How did you get involved? Were you referred? By whom?

Let’s talk about your first class. What stands out for you about that experience?

How would you describe your level of participation in the exercise classes? Did you find the classes engaging?

How would you describe the communication you had with the instructor? Were you able to interact with other class participants?

Was there anything you particularly liked about the cardiac rehabilitation class?

For example, was it the exercises/the instructor/the group dynamic/?

Was there anything you didn’t like about the class?

The other group of participants in this study partook in online exercise classes, joining their exercise class via Zoom, twice per week.

- What would your thoughts be online exercise classes? Would it be something you’d do?
- Would you feel comfortable/safe doing the classes online? Why/Why not?
- Do you think there are any benefits to doing these classes online in your own home?
- Do you think there are any drawbacks to doing these classes online in your own home?

**Health Related Questions**

How would you describe your health since participating in the study?

**Prompts:**

For example, has your health improved, declined or remained the same?

What are the reasons you think your health has improved, declined or remained the same?

How would you rate your physical fitness?

**Prompts:**

Has your fitness improved, declined or remained the same?

How do you feel about exercise after taking part in the classes?

Are you more inclined to work some form of physical activity or exercise in to your daily schedule now?

**Conclusion**

Would you recommend this type of cardiac rehabilitation to a patient with similar needs as you?

**Prompts:**

Can you explain why you would or would not recommend?

Is there anything else that you would like to comment on that we haven’t discussed today?

Thank you very much for your time and the information you shared today.
